# Beyond Sex: Gender, LGBTQ, and Alzheimer’s Disease and Related Dementias

**DOI:** 10.1093/geroni/igaa057.2692

**Published:** 2020-12-16

**Authors:** C Elizabeth Shaaban, Michelle Mielke

## Abstract

Sex and gender are important sources of variation in Alzheimer’s disease and related dementias (ADRD) and associated caregiving. Women comprise 2/3 of ADRD cases and the majority of ADRD caregivers. Sex encompasses biological differences due to sex chromosomes, reproductive tract, and hormones, while gender constitutes socioculturally constructed psychosocial aspects of sex. Several lines of research have begun to interrogate sex differences, but less is known about the relation of gender and lesbian, gay, bisexual, transgender, and/or queer (LGBTQ) status with ADRD. In this symposium featuring both trainees and faculty we highlight novel research addressing these factors from multiple perspectives. Two presentations address how psychosocial characteristics and their strengths of association with brain health may vary by gender. C. Elizabeth Shaaban presents analyses testing whether gendered psychosocial factors explain sex differences in white matter hyperintensities, a neuroimaging marker of cerebral small vessel disease and risk factor for ADRD. Justina Avila-Rieger presents results testing region of birth-based spatial patterning of dementia risk among Black men and women. Next, Jason Flatt presents prevalence estimates of subjective memory problems and dementia and describe factors associated with dementia among LGBTQ older adults. Finally, gender may also impact perceptions of individuals with dementia. Shana Stites explores gender differences in AD stigma and discuss implications for who is willing to be an AD caregiver. Michelle Mielke, an expert in sex and gender differences in neurodegenerative and age-associated diseases will facilitate conversation about these results and place them in the context of current sex and gender-based ADRD research.

